# Clothing and Outdoor Thermal Comfort (OTC) in tourist environments: a case study from Porto (Portugal)

**DOI:** 10.1007/s00484-024-02753-y

**Published:** 2024-09-02

**Authors:** Hélder Silva Lopes, Paula C. Remoaldo, Vítor Ribeiro, Javier Martín-Vide, Inácio Ribeiro

**Affiliations:** 1 Lab2PT - Laboratory of Landscape, Heritage and Territory/IN2PAST - Research and Innovation in Heritage, Arts, Sustainability and Territory/Department of Geography /ICS, University of Minho, Guimarães, Portugal; 2 IdRA - Water Research Institute/Climatology Group/ Department of Geography/FGH, University of Barcelona, Barcelona, Spain; 3 ESE de Paula Frassinetti, Porto, Portugal; 4 Department of Geography/ICS, University of Minho, Guimarães, Portugal

**Keywords:** Outdoor thermal comfort (OTC), Clothing insulation (Icl), Tourist Perception

## Abstract

This study focuses on assessing tourists' perception of bioclimatic comfort in the urban context of Porto, Portugal, specifically in the areas of Avenida dos Aliados and Praça da Liberdade. The study examines the relationship between meteorological conditions, tourists' clothing choices, and their physical activity levels. The study integrates microclimatic measurements and questionnaire surveys carried out during the summers of 2019 and 2020, and the winter of 2019-2020. A comprehensive questionnaire following international standards was administered to a representative sample of 563 tourists. The results show significant variations in mean air temperature (AT), wind speed (Wχ), relative humidity (RH), global radiation (G_RAD_), and total mean radiant temperature (T_MRT_) over the study periods. The assessment of Outdoor Thermal Comfort (OTC) is based on ASHRAE 55 standards, using the Thermal Sensation Vote (TSV) scale and the tourists' opinions on their thermal preferences. Clothing choices are found to be influenced by AT, with tourists choosing lighter clothing in warmer conditions. Gender and age differences in clothing insulation (Icl) are identified, suggesting potential differences in OTC perception. AT varied significantly, with an inflection point in clothing choices at 21.7°C and a correlation between AT and reduction in clothing layers (r^2^ = 0.846; *p* < 0.05). The study also observes seasonal variations in physical activity levels of tourists, with higher activity levels in summer due to milder weather (110.0 W·m⁻^2^). More thermally comfortable environments tend to promote a sense of well-being among visitors, which directly affects their satisfaction during their stay in the city. When tourists feel comfortable with the thermal conditions of the urban environment, they are more likely to explore and enjoy local attractions for longer periods of time, thereby enhancing their cultural and leisure experiences. Women tend to wear fewer layers of clothing than men in summer, reflecting potential differences in OTC perception. Results align with previous studies, indicating the impact of clothing insulation of individual subject (Icl) on OTC varies across locations and cultures. Cultural factors influence clothing preferences and thermal tolerance, emphasizing the need for nuanced considerations in understanding OTC perceptions. The study provides to the understanding of the OTC of tourists in the city of Porto, but also offers relevant contributions for improving the visitor experience and sustainable development, namely in other geographical contexts. The major contribution of this research lies in the comparative analysis of Icl and OTC between tourists, based on physical measurements and questionnaire surveys conducted in summer and winter, providing valuable insights for tourist spot design.

## Introduction

Clothing plays a crucial role in the adaptation of individuals to outdoor spaces, especially in less favorable weather conditions (Salata et al. 2018). In addition to serving as insulation between the human body and the external environment, clothing is intrinsically linked to the culture or characteristics of a particular society. The resistance to heat exchange, known as the thermal insulation of clothing, is often expressed in Icl, where one Icl is equal to 0.155m^2^ºC/W (Zafarmandi et al. 2024). The amount of thermal insulation used by an individual plays a significant role in OTC (Environmental Thermal Conditions for Human Occupancy, 2010), according to American (ASHRAE 2010), European (European Committee for Standardization - CEN, 2018), and international rules for calculating Thermal Sensation Vote (TSV - ISO 7730, 2005).

It is important to remember that OTC is significantly influenced by microclimate conditions and personal parameters. The variety of clothing used in different regions reflects the different climatic conditions. Outdoor thermal conditions are transient, with wind frequency and speed as well as temperature changes playing a significant role in triggering thermal sensation overshoots and better comfort than equivalent indoor conditions (Ma et al. 2019). Similarly, individuals adjust the clothing worn according to the ambient temperature, reducing layers in warmer climates, and adding layers in colder climates to achieve OTC (Lai et al. 2017; Nikolopoulou & Steemers 2003; Van Hoof & Hensen 2007). Human responses to thermal environments include self-adaptive and communal adaptive behaviors. Self-adaptive behaviors include individual behavioral changes such as clothing choice, posture, and activity level, while communal adaptive behaviors include actions such as opening windows and using umbrellas (de Dear et al. 1997; Nikolopoulou & Lykoudis 2006; Mohan et al. 2013; Lai et al. 2014; Lopes et al. 2021b; Liu et al. 2023).

As the climate-weather changes, so do our clothing choices. In hot weather, we shed layers, while cooler temperatures prompt us to bundle up. This self-regulation, as described by Lai & Chen (2017), Nikolopoulou & Steemers (2003), and Van Hoof & Hensen (2007), allows us to maintain a comfortable internal temperature despite external fluctuations.

However, in certain situations, such as the workplace, academic settings, or cultural traditions, dress codes and social norms may limit clothing choices. Cultural influences also play a role, as explored in this study. Recent studies such as Salata et al. (2018) suggest that women and older adults in the Mediterranean region tend to wear more clothes.

In fact, few studies have examined the effect of Icl on subjective thermal perception and comfort, as mentioned in Zafarmandi et al. (2024). Understanding Icl is critical to accurately assessing thermal comfort and designing spaces that promote well-being.

Therefore, when designing or evaluating the outdoor thermal environment, it is crucial to consider the specific needs of different groups of people, such as gender and age, and to understand their responses to the thermal environment, namely clothing insulation of individual subject (Icl), activity level, and adaptive behaviors (Lin & Matzarakis 2011). This study aims to analyze people's responses to the outdoor environment in tourist locations, focusing on differences in clothing preferences and activity states. Previous research has focused on people's clothing. However, our study is unique in that it focuses on the influence of clothing thermal insulation on outdoor OTC and sensation in Porto, Portugal.

This paper is organized in six sections. Following this introduction, Section 2 presents the literature review, which deals with the theoretical contribution to the analysis of OTC in tourism. Section 3 also reviews the literature related to the effect of clothing use on OTC during a tourist trip. Then, section 4 presents the methods used for data collection and analysis, while the results, in section 5, highlight the main relationships between microclimatic measurements and clothing adjustments and OTC. In section 6, the discussion and conclusions interpret the results in both theoretical and practical contexts, summarizing the contributions of the study.

## Theoretical Contributions on Outdoor Thermal Comfort (OTC) Analysis in Tourism

The analysis of OTC emerges as a highly relevant tool in the field of tourism, representing 4.0% of the studies dedicated to the promotion of bioclimatic comfort in tourist destinations, as identified by Kumar & Sharma (2020), covering the period from 2001 to 2019. The study of OTC originated in the early 20th century, pioneered by Gagge (1936), who studied the interaction between human body temperature and the environment (Remoaldo et al. 2023). Fanger (1970) defined OTC as a state of neutral thermal sensation or thermal equilibrium. The American Society of Heating, Refrigerating, and Air-Conditioning Engineers (ASHRAE 2013) considers OTC as a mental state that expresses satisfaction with the thermal environment. This concept is subjective, resulting in multiple definitions. While initially associated primarily with indoor environments (Fabbri 2024), studies on OTC have expanded to include outdoor environments over the past six decades.

It's important to note that, despite everything, there are few studies that evaluate the effects of wearing certain clothing in destinations (Rozbicka & Rozbicki 2021, 2023; Lindner-Cendrowska 2013; Tian et al. 2022; Xi et al. 2020; Zafarmandi et al. 2024 – Table 1).

**Table 1:** Key studies on outdoor thermal comfort analysis of tourists

Study	Geographic location	Köppen-Geiger classification	Sample (n)	Analysis method	Factors considered in analysis	Thermal comfort evaluation	Tourist segment	Time scale
Lindner-Cendrowska (2013)	Warsaw, Poland	*Cfb*	553 tourists	Frequency, distribution, Regression	Physical parameters	AT, Icl, UTCI, PET, TSV, TPV	Urban	July 2010, February 2011, April and October 2011
Rutty & Scott (2015)	Caribbean (tourists from Canada)	*Am*	216 tourists	Regression, frequency, ANOVA test	Physical parameters	AT, W, UTCI, TSV, TPV	Sun and beach	March-April 2012
Kariminia et al. (2016a, 2016b)	Isfahan, Iran	*Bsk*	504 tourists	Non-linear model (Neural Network Autoregressive with Exogenous Input - NN-ARX)	Physical parameters, square location, age, sex, activity, nationality	Tg, AT, RH - %, I, W, PET, PMV, SET, T_MRT_, TSV	Urban	July 12-24, 2014
Kovács et al. (2016)	Szeged, Hungary	*Dfb*	5 128 tourists	Frequency, description, Regression	Physical parameters	PET, TSV, TPV, TCI, CTIS	Urban	Summer, autumn, winter 2011 and 2012
Nasrollahi et al. (2017)	Isfahan, Iran	*Bsk*	281 tourists	Frequency, description, Regression, spatial modeling	Physical parameters, exposure time, mental conditions of tourists	AT, RH - %, n, I, Wind, T_MRT_, PET, SET, PMV, ET, OUT-SET, UTCI, TSV, TPV	Urban	July 2016
Lindner-Cendrowska & Błażejczyk (2018)	Varsóvia, Polónia	*Cfb*	662 tourists	Frequency, description, Regression	Physical parameters, sex, age, air conditioning (yes or no), nationality, climatic origin according to Köppen-Geiger	AT, RH - %, I (globe), W, T_MRT_, PET, TSV, TPV	Urban	July 2010, February 2011, April and October 2011
Xi et al. (2020)	Harbin, China	*Dwa*	1740 tourists	Frequency, description, Regression	Physical parameters, physical activity, clothing	AT, RH - %, W, I (globe), Icl	Urban	Winter and summer, between December 2017 and January 2019
Lopes et al. (2021a, b)	Porto, Portugal	*Csb*	563 tourists	Frequency, description, Regression	Physical parameters, sex, age, air conditioning (yes or no), nationality, climatic origin according to Köppen-Geiger	AT, RH - %, I, W, T_MRT_, PET, TSV, TPV	Urban	Summer 2019, Winter 2019 – 2020 and Summer 2020
Karimi & Mohammad (2022)	Sevilla and Madrid, Spain	*Csa and BSk*	180 tourists	Frequency, description, Regression	Physical parameters	AT, W, UTCI, TSV, TPV, T_MRT_, PET, SET	Urban	July 2021
Tian et al. (2022)	Xi'na, China	*Dwa*	1772 tourists	Frequency, description, Regression	Physical parameters, physical activity, clothing	AT, RH - %, W, I, TSV, TPV, Icl	Urban	Winter (January 1–3, 2021), spring (April 16–18, 2021) and summer (July 24–26, 2021)
Rozbicka & Rozbicki (2021, 2023)	Warsaw, Poland	*Cfb*	776 tourists	Frequency, description, Regression	Physical parameters, exposure time, mental conditions of tourists	AT, RH - %, PET, TSV, TPV W, I (globe), Icl	Urban	Summer, between June to August 2019
Zafarmandi et al. (2024)	Tehran, Iran	*Bsk*	838 tourists	Frequency, description, Regression	Physical parameters, physical activity, clothing	AT, W, UTCI, TSV, TPV, T_MRT_, I(globe), PET, Icl	Urban	March 2019 to February 2020

Source: Own elaboration

*Icl* Clothing insulation, *CTIS* Climate Tourism Information Scheme *RH* % Relative humidity, _*I*_ Insolation [I(globe) – insolation extracted from the black globe], *OUT-SET* Outdoor Thermal Comfort Index, *PET* Physiological Equivalent Temperature, *SET* Standard Effective Temperature, *AT* Air temperature, *ET* Efective temperature, *Tg* Globe temperature, *T*_*MRT*_ Mean Radiant Temperature, *TPV* Thermal Preference Vote, *TSV* Thermal Sensation Vote, *W* Wind, *UTCI* Universal Thermal Comfort Index

For the conduct of the research, the use of theories to investigate and explain the patterns found in OTC is relevant. This is because different geographical contexts are associated with different climatic-meteorological conditions and, consequently, perceptions of outdoor OTC and the use of public space in different urban planning and design contexts (Ma et al. 2021).

Although various models have been developed for the analysis of OTC and have been used in the design of urban spaces and the provision of more pleasant urban spaces, it is widely recognized that the state of comfort can be influenced by various qualitative parameters that cannot be assessed by physical models of thermal balance (Brager & De Dear 1998; Thapa & Indraganti 2020; Vasilikou & Nikolopoulou 2020).These parameters include:

(i) Sociodemographic factors, namely gender, age, culture, or socioeconomic status (Aljawabra & Nikolopoulou 2010).
(ii) Contextual parameters, depending on the type of construction, activities in the space, climate, and season (e.g., Chen & Ng 2012; Ng 2012).
(iii) Multisensory and environmental interaction with the space (including thermal, auditory, visual, and olfactory dimensions – e.g., Henshaw 2013; Vasilikou 2018; Vasilikou & Nikolopoulou 2020).
(iv) Cognitive parameters (use, preferences, and expectations – e.g., Nikolopoulou & Lykoudis 2006; Lenzholzer and Koh 2010; Klemm et al. 2015; Lam et al. 2018, 2021).
(v) Information that the tourist has predefined about a specific environment (Nikolopoulou et al. 2001).
(vi) Sensory experience over an urban continuum, where the transition between spaces includes a mix of pleasant-unpleasant experiences and the body adjusts based on movement characteristics, evaporative cooling (sweat production), and metabolic rate. This parameter integrates the Icl.

These parameters are crucial for the adaptability and thermal satisfaction in the open air (Baker 2001), and it is essential to consider the psychological factors inherent in a previous experience (Nikolopoulou et al. 2001), associated expectations and information obtained through various means (Knez & Thorsson 2006) to determine the comfort level in a space. This last condition will be essential for the decision to use a greater or lesser number of items of clothing.

## Effect of clothing use on Outdoor Thermal Comfort (OTC) during a tourist trip

The Icl is strongly correlated with individual habits and one's country of origin. Therefore, a regional characterization of standard preferences regarding the thermal resistance of clothing (expressed in a statistically significant sample of individuals with certain characteristics) is essential for understanding this theme. It is crucial to highlight a range of limits within which OTC levels (or neutrality) can be achieved by adjusting the Icl, considering different seasons of the year (Camacho 2018; Wang et al. 2019).

Exchanges between the skin surface and the atmosphere are modulated by clothing, acting as a new surface that interferes between the skin and the environment.

The magnitude of each depends on incident flow and the color and density of the fabric. Most clothing is designed to protect the body against excessive loss of sensible heat, managed through the choice of clothing in line with the insulation level of various pieces (Varadaraju & Srinivasan 2019). Note also that values change according to the nature (e.g., GoroTex, HydroTex) and the extent of the body they cover (e.g., Mokhtari Yazdi & Sheikhzadeh 2014; Golasi et al. 2018; Rosso et al. 2018; Varadaraju & Srinivasan 2019). The use of specific clothing sets during different seasons depends on the individual characteristics of the tourist and their country of origin. However, there are some that are typically more used in the summer, such as short-sleeved shirts/t-shirts, shorts, and dresses (with a predominance of fabrics with special thermal properties that control perspiration – GoroTex and HydroTex), or in the winter with thermal clothing, coats, gloves, and hats (Golasi et al. 2018; Zafarmandi et al. 2024 – Table 2).

**Table 2:** Isolation values (Icl) for individual clothing items or sets of clothing

**Clothing items**	**Isolation Icl (K m**^**2**^** W**^**-1**^**)**	**Stable Air Layer Depth (mm)**
Individual clothing items		
* Underwear (e.g., briefs, boxers)*	0.03-0.10	0.15-0.52
* Footwear (e.g., socks, slippers, boots)*	0.02-0.10	0.10-0.52
* Shirts/Blouses (e.g., short-sleeved sweater, long-sleeved shirt)*	0.15-0.30	0.78-1.55
* Pants (e.g., jeans, overalls)*	0.06-0.28	0.31-1.45
* Sweaters/Sweatshirts/Jackets*	0.20-0.35	1.03-1.81
* Dresses/Skirts*	0.15-0.40	0.78-2.07
* Coats/Parkas*	0.55-0.70	2.84-3.62
Sets of clothing		
* Long-sleeved underwear and legs, shirt, pants, jacket, socks, and shoes*	0.155	5.17
* Short-sleeved underwear and legs, shirt, pants, jacket, thermal coat and pants, socks, and shoes*	0.225	7.50
* Long-sleeved underwear and legs, thermal coat and pants, padded parka, socks, shoes, hat or cap, and gloves*	0.395	13.17

Source: Adapted from Parsons (2014)

1 mm of stable air depth corresponds to ≈ 3,85· 10^-2^ K m^2^ W^-1^

The human energy balance results from the overall effect of sets of clothing (e.g., shoes, socks, underwear, pants, shirts) that cover practically the entire person (Qian & Fan 2009; Oliveira et al. 2011). The clothing commonly worn reflects the individual's climatic environment, although there are places where customs and traditions influence the use of specific clothing, often interfering with the clothing a tourist will use during their visit to a country (Brager & De Dear 1998; Niinimäki 2010). On the other hand, we can't forget the influence of fashion on the choice of clothing in tourist destinations.

International standards also describe numerical calculations to obtain the thermal resistance of clothing and should be used for this purpose (e.g., ISO 10551 1995; VDI 2008; ISO 8996 2004; ISO 7730 2005; ISO 2006; ISO 9920 2007; ASHRAE 2013, 2017). Altogether, the available information on Icl, although not providing specific indications, is relevant for assessing bioclimatic comfort. In addition to these characteristics, consideration should be given to the origin of the population (and the associated climate type), age, and gender. Having this information is relevant to contribute to filling gaps that exist among stakeholders when managing the destination or making recommendations for places to visit and what prerequisites to fulfill.

The response to cold and heat is manifested in the degree of comfort with the thermal environment, which is essentially a subjective assessment (ASHRAE 2013, 2017). In this sense, tourists feel comfortable when they do not want to change their clothing layers and/or activities in the environment to which they are exposed because they do not feel hot or cold (Lopes et al. 2021b; Rutty & Scott 2015). It should also be noted that deviations from comfort produce sensations of heating or cooling, depending on biophysical parameters (e.g., sweat rate or skin temperature) and individual preferences [depending on cultural context, thermal expectations, and level of acclimatization] (Grigorieva 2021; Lin & Matzarakis 2011; Zafarmandi et al. 2024).

## Methods and data

This case study, applied in Avenida dos Aliados and Praça da Liberdade in Porto, analysis is based primarily on a subjective dimension of the research, which includes tourists' perceptions of OTC in urban tourist environments. This information was collected through a questionnaire survey administered to tourists. In addition, microclimatic measurements were carried out to assess the perception of bioclimatic comfort in public spaces and the enjoyment capacity of the places visited, ensuring the integration of the objective evaluation.

### Study area

**Fig. 1:**
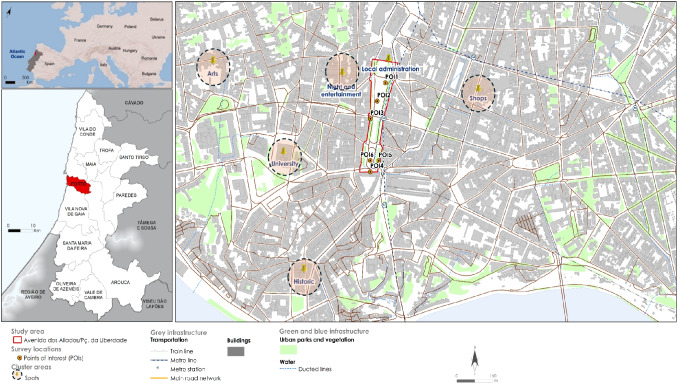
Case study area – Avenida dos Aliados and Praça da Liberdade (Porto). **A** European view; (**B**) Porto Metropolitan Area; and (**C**) Location of study area. Source: Own elaboration

The study was conducted in the areas of Avenida dos Aliados and Praça da Liberdade, two of the most visited areas in Porto. The city, located in the northwest of Portugal, covers an area of 41.42 km^2^ and is part of the Porto Metropolitan Area. Porto is internationally recognized for its coastal location, temperate maritime climate, and economic dynamism (Fig. 1).

Porto experiences occasionally warm summers, reaching up to 35ºC, and rainy and cool winters, with temperatures between 5ºC and 14ºC. According to the Köppen-Geiger climate classification, Porto falls into the Csb zone. The city has gained international recognition as a quality tourist destination (Lopes et al. 2021b).

By 2023, the number of tourists will have increased, establishing Porto as one of the most visited cities in the world. Tourist growth has been influenced by media coverage and the valorization of new urban spaces.

So far, the number of tourists has increased, recording more than 2,700,000 overnight stays in 2023 according to INE, establishing Porto as one of the most visited cities in the world. Furthermore, Porto won several awards within the scope of the "Tourism Oscars", such as: Best Metropolitan Seaside Destination 2024; Europe's Leading City Break Destination 2023; Best City Destination in the World 2022; between others.

Porto, a "Tourist Historic City", has been investing in the promotion of tourism since 2014 (Lopes et al 2021a; 2022). In addition, programs have been implemented to adapt the city to climate changes and improve OTC in different urban spaces, such as measures from the Municipal Strategy for Adaptation to Climate Change (EMAAC) (CMP 2024). This municipal strategy aims to mitigate climate impacts through the installation of green roofs, the LIFE-myBuildingisGreen project co-funded by the European Union (EU) to evaluate and monitor the effectiveness of nature-based solutions (NbS) in improving the bioclimatic comfort of buildings, or the Native Urban Forests project, which aims to produce native trees and shrubs for planting in strategic locations in the city.

It is certain that the changes made in the city center of Porto have modified thermal, hygrometric, and anemometric characteristics, contributing to intra-urban differentiation compared to neighboring spaces (Monteiro et al. 2018). These individual characteristics translate into different bioclimatic comfort conditions due to the urban artificialization and volumetrics created, in addition to the primary physical conditions (elevation, distance to water bodies, solar exposure, or slope). Even in small areas like Avenida dos Aliados, the characteristics can be quite different.

### Microclimatic measurements

Experiments were conducted with the assistance of senior technicians in architecture and civil engineering. First, a Computational Fluid Dynamics (CFD) simulation was performed to provide an initial indication of the summer and winter wind flows within the Avenue/Plaza. This involved calibrating wind impact studies by introducing four parameters into the CFD simulation: prevailing wind direction, wind speed (Wχ), building height, and horizontal plane in the simulation area. This analysis is made with the use of Envi-Met and AutoCAD software.

Concerns were raised about the possibility of a wind tunnel effect and higher wind speeds over the intermediate area of Aliados (Charalampopoulos et al. 2017; Fröhlich et al. 2019; Nouri & Costa 2017). Therefore, it was decided to use lower wind speeds than those recorded by meteorological stations, with values of 3 m.s^-1^ for summer and 6 m.s^-1^ for winter, based on previous studies of daily wind regimes in Porto.

Subsequently, a second simulation aimed to analyze the solar radia tion within Avenida dos Aliados and Praça da Liberdade using Shadow Behavior Simulations (SBS). Due to the uniform building heights in the Avenue, a height of 18 m was used in the model.

These morphological considerations were important, especially considering the relatively low height-to-width (H/W) ratio of the buildings in the study area compared to the surrounding areas. Each simulation was calibrated to achieve the desired period and accuracy, with the first study focusing on a complete daytime analysis of Avenida dos Aliados and Praça da Liberdade. After the experimental procedures in the laboratory, measurements were taken in the field using four identified instruments. These instruments were calibrated by the manufacturers to ensure accuracy during the study interval.

We followed the recommendations of ISO 7726 (2006) and the ASHRAE manual for instrument configuration, measurement range, and accuracy. The four instruments used to analyze wind speed (WS), ambient temperature (AT), relative humidity (RH), global radiation (GRAD), and surface temperature (ST) are shown in Table 3. All instruments were positioned at a height of 1.1 meters. For AT and RH measurements, we avoided direct sun exposure to avoid overestimation of AT. We used sensor protection against radiative exchange between the instrument and the environment. In addition, we allowed approximately 1.5 minutes for the sensor response time before starting the measurement, considering the thermal inertia of the instrument.

**Table 3:** Equipment used during fieldwork

	**Equipment name/model**	**Measurement**	**Unit of measurement of the *output***	**Accuracy**	**Resolution**
1		*AT RH*	ºC %	± 0,5ºC (AT) 3% ±0.1	0,1ºC 0,1%
2		_*AT*_*WxFlow*	ºC m.s^-1^--	±1 ±3% ±0.1 calculated from wind speed and surface area	0,01m.s^-1^
3		*G*_*RAD*_	W.m^-2^	±1	0,1 W.m^-2^
4		*T*_*surf*_	ºC	1.5%	0,1ºC

Source: Own elaboration

The sensors complied with ISO 7726 for Wχ and direction. The time interval between measurements was sufficient to cover the difference between low and high wind speeds. We calculated the Wχ by combining two parameters: the maximum speed recorded during the survey (Wχ) and the standard deviation (SD) of the wind recorded during the same period. For the Mean Radiant Temperature (T_MRT_), which was calculated by modeling using the RayMan software, we followed the proposed methodologies of previous studies (Crank et al. 2020; Matzarakis et al. 2010). We combined collected and recorded data in the software. The RayMan-based simulation required input variables such as day and time information, geographic location, urban structure and environmental morphology, Sky View Factor (SVF) based on fish-eye image usage, and meteorological data.

Data collection was based on several assumptions, including instrument configuration and accuracy, protection of sensors from direct sunlight to avoid temperature overestimation, and sufficient time between Wχ measurements to cover differences.

**Fig. 2:**
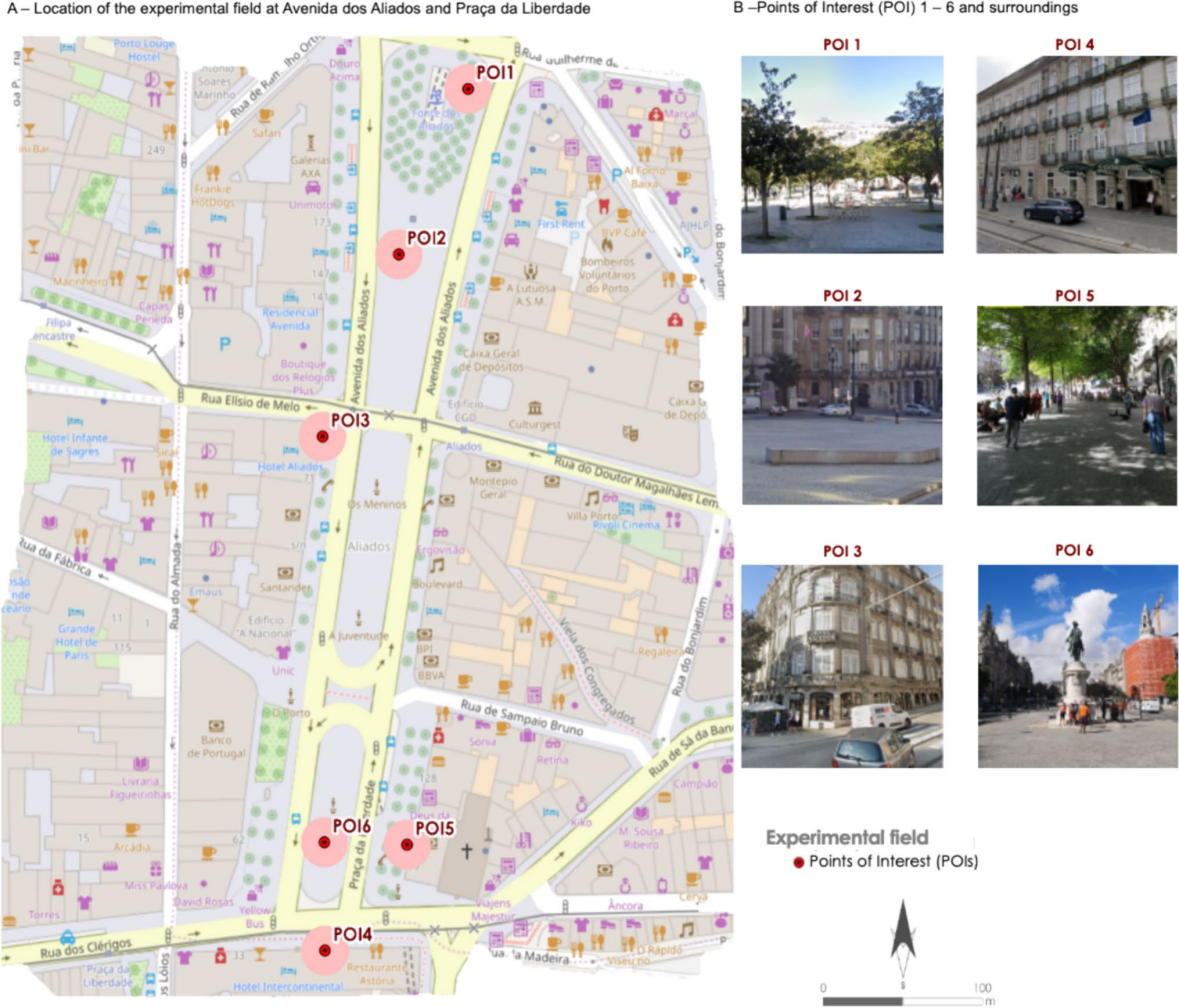
The typology of outdoor contexts in Points of Interest used during the fieldwork. **A** Location of the experimental field at Avenida dos Aliados and Praça da Liberdade; and (**B**) Points of interest (POI) 1 – 6 and surroundings. elaboration and photos taken by authors. Source: Own elaboration and photos taken by authors

Six analysis points were considered for data collection (see Fig. 1). This allowed the recording of diurnal microclimatic variations in the study area. The selection of these six points was based on the characteristics associated with the different areas under analysis (proximity to water; with vegetation; without vegetation; in the center of the avenue). It was also taken into account that the Avenida dos Aliados is usually frequented by people of different ages, mostly visited by tourists, and associated with transportation, cafes, restaurants and supermarkets. The typology of outdoor contexts was explained through illustrations in Fig. 2.

Measurements were made over a period of eight days for summer 2019, winter 2019-2020 and summer 2020 (see their characteristics and weather patterns in Table 4), focusing on days with stable conditions and under the influence of the Azores Anticyclone. On a recurring basis, the prevailing winds were mainly NNO, although on the days of the winter study, the wind direction was more irregular (mainly ESE-E). These characteristics are obtained from the Portuguese Institute of Ocean and Atmosphere (IPMA).

**Table 4:** Atmospheric conditions for the days of the survey

**Day**	**AT (Max) (ºC)**	**AT (Min) (ºC)**	**RH (%) max**	**RH (%) min**	**Wind (MpH) max speed**	**Prevailing wind direction**	**Type of atmospheric circulation**	**Nebulosity conditions**
July 14, 2019	22	17	100	78	9	WNW- NW	Depression regions centered west of mainland Portugal or of thermal origin over the Iberian Peninsula	Partly/ Mostly cloudy
July 21, 2019	24	13	100	69	10	NNW- NW	Anti-cyclone located southwest or over the Azores Archipelago, sometimes extending ridge to the British Isles or with multiple cores, in action combined with thermal depression over the eastern part of the Iberian Peninsula	Fair
August 15, 2019	24	15	100	57	11	NNW-NW	Anti-cyclonic crest, associated with a high pressure center whose location oscillated in the Atlantic between the northeast and southwest regions of the Azores archipelago. Thermal depression, centered in the interior of the Iberian Peninsula	Fair
August 21, 2019	28	17	94	47	10	NNW	Anti-cyclonic crest, associated with a high pressure center whose location oscillated in the Atlantic between the northeast and southwest regions of the Azores archipelago. Thermal depression, centered in the interior of the Iberian Peninsula	Fair
December 24, 2019^1^	19	12	100	68	11	ESE - E	Blocking anticyclone over the Iberian Peninsula, North Africa and Western Europe. East quadrant current	Fair
December 28, 2019^2^	19	8	81	32	12	ESE - E	Blocking anticyclone over the Iberian Peninsula, North Africa and Western Europe	Fair
February 23, 2020^3^	20	5	100	49	4	WNW - NNW	Anti-cyclone located in the Iberian Peninsula or in the Azores extending along the ridge towards the peninsula	Fair
July 11, 2020	30	16	94	40	10	VAR (namely, NNW – NW – WNW)	Anticyclone located over the British Isles. Approaching or passing through low activity frontal surfaces	Fair
August 05, 2020	25	13	100	57	13	NNW - NW	Anti-cyclone located in the region of the Azores archipelago, extending into the crest to the Bay of Biscay or to the British Isles. Thermal depression and/or inverted valley extending from northern Morocco to the Iberian Peninsula	Fair
August 08, 2020	23	16	100	73	9	S	Anti-cyclone located in the region of the Azores archipelago, extending into the crest to the Bay of Biscay or the British Isles	Fog/ Mist
August 13, 2020	22	13	100	68	9	NNW – WNW	Anti-cyclone located over the Azores archipelago	Fair/ Partly cloudy
August 21, 2020	23	16	100	69	9	VAR (namely, SE, SSW, SW)	Anticyclone located in the Azores archipelago, or south of it Depressions centered to the west or on the Iberian Peninsula, with expression in altitude and / or approaching or passing frontal surfaces	Partly cloudy/ Fair

Source: Own elaboration

Important events: ^1^Christmas Eve; ^2^S. Silvestre Running; ^3^Carnival Sunday

### Questionnaire survey on tourists’ perception of bioclimatic comfort

#### Questionnaire structure

The questionnaire used in the research was divided into three sections (A - C), with a total of 27 questions divided into sub-items (see Appendix 1 for details). The questionnaire followed a conventional structure, progressing from global to more specific questions. It was designed based on international standards and aligned with questionnaires used in similar investigations (e.g., Spagnolo & De Dear 2003; Scott et al. 2008; Lindner-Cendrowska 2013; Rutty & Scott 2015; Kovács et al. 2016; Lindner-Cendrowska & Błażejczyk 2018; Amininia et al. 2020; Xi et al. 2020).

The questionnaire consisted of three main sections: (1) travel experience in the AMP (Área Metropolitana do Porto), (2) climatic-meteorological experience in Porto [with emphasis on AT, RH (%), Wχ, cloudiness, and solar radiation], and (3) socio-demographic characteristics. It is important to note that in this research, beyond purely climatic perceptual parameters such as temperature, precipitation, wind and cloudiness, the focus was on the evaluation of OTC. For this purpose, the research was based on the ASHRAE 55 standards (ASHRAE 1992) and ISO 10551 (1995), specifically using the TSV scale on a 7-point Likert scale [very cold (-3), cold (-2), slightly cold (-1), neutral (0), slightly warm (1), warm (2), and very warm (3)] and the tourists' opinions on their thermal preferences on a 5-point scale (considering the AT variable) (Appendix 1).

To assess the sociodemographic context of the surveyed tourists, a section was dedicated to recording age, gender, education level, weight, height, and analyzing the activity level and clothing in accordance with ISO 8996 (2004) and ISO 9920 (2007), respectively. At the beginning of the questionnaire, we explained the purpose of the study and how it would be useful in assessing the thermal comfort of tourists. Personal data has been anonymized and explained to participants in strict compliance with European data protection legislation, namely Regulation EU 2016/679 and the corrigendum published in the Official Journal of the EU on 23 May 2018 on the protection of personal data and on the free movement of such data (European Commission, 2023). For each survey, AT, RH (%), and Wχ were recorded at the time of the questionnaire application and noted in questionnaire. GRAD and ST were recorded on a data sheet attached to each questionnaire. Microclimatic measurements were taken every 90 seconds, concurrently with the surveys. Between 3 and 4 measurements were taken during each survey. The questionnaire was estimated to take approximately 10 minutes to complete.

#### Sample selection and result analysis

The age range was limited to 15 years or older. The selection also considered the cognitive maturity recommended by other previous studies (Valle et al. 2012; Remoaldo et al. 2014; Lopes et al. 2021a). Since it was not possible to survey all tourists to Porto, a representative sample was selected using data from guests in 2018. Simple random sampling was used to determine the sample size, considering the number of tourists, time, and cost. To achieve 95% confidence, a sample of 385 individuals was targeted, representing 0.02% of guests in 2018 listed by the Porto and Norte Tourism Entity. This sample size was considered sufficient to compare tourists' perspectives with instantaneous meteorological parameter measurements, especially if it considers other realized studies (see Table 1).

Ethical review and approval were not required for this study. The project was approved in September 2017 (funded by FCT Portugal, grant number SFRH/BD/129153/2017 and approved with reference ICS-120/2017). This project fell outside its scope, and thus, ethics committee approval was not necessary. Informed consent was obtained from all subjects involved in the study when the questionnaire survey was completed.

Pretesting plays a critical role in research by anticipating data collection and identifying limitations. The evaluation focused on assessing average response time, clarity of questions, optimal order, and the need for additional questions (Lopes 2016; Brace 2018; Lopes et al. 2019). The goal was to obtain initial feedback, check the validity of the constructs, and ensure the reliability of the results to ensure that they accurately reflected the concept being assessed. This exercise proved to be very useful as it allowed for the elimination of questions and the estimation of individual response time within a maximum of 7 minutes. All questions and responses from the research were coded, entered, and analyzed using the Statistical Package for the Social Sciences (SPSS). This program uses descriptive and inferential statistical tools to analyze quantitative data. Statistical analyses were performed at a 95.0% confidence level (*p* <0.05).

#### Sample Characterization

This study was based on 563 valid surveys distributed over three different periods: summer 2019 (36.8%), winter 2019-20 (25.9%), and summer 2020 (37.3%). The sample included 278 males (49.4%) and 285 females (50.6%), covering different age groups, with the majority (67.7%) falling between the ages of 25 and 44 (Table 4). In terms of travel planning, 43.9% of participants planned their trips between 1 and 5 months in advance, while most visits lasted 2 to 3 days (50.4%), highlighting the profile of city breaks, in line with previous studies on Porto (e.g., Lopes et al., 2021b)  (Table 5).

**Table 5:** Participants' responses to demographic, physiological and psychological variables included in the questionnaire used in the research

**Variables**	**Summer 2019 **(*n*=207)		**Winter 2019-2020 **(*n*=146)		**Summer 2020 **(*n*=210)		**ANOVA**		**Total **(*n*=563)	
	**n**	%	n	%	n	%	F	*p-value*	n	%
***Gender***										
**Male**	107	51,7	72	49,3	99	47,1	0,430	0,651	278	49,4
**Female**	100	48,3	74	50,7	111	52,9			285	50,6
***Age***										
15-24	22	10,6	17	11,6	20	9,5	0,807	0,447	59	10,5
25-44	146	70,5	94	64,4	141	67,1			381	67,7
45-64	31	15,0	30	20,5	44	21,0			105	18,7
65 and over years	8	3,9	5	3,4	5	2,4			18	3,2
***Country of residence***										
Portugal	35	16,9	22	15,1	43	20,5	12,346	0,000**	100	17,8
**Another European country**	106	51,2	74	50,7	144	68,6			324	57,5
**America**	20	9,7	19	13,0	1	0,5			40	7,1
**Another continent**	46	22,2	31	21,2	22	10,5			99	17,6
***Education***										
Less than 6 years of schooling	7	3,4	4	2,7	3	1,4	1,117	0,328	14	2,5
7th - 9th grade	20	9,7	13	8,9	16	7,6			49	8,7
10th - 12th grade	70	33,8	50	34,2	64	30,5			184	32,7
Graduation	81	39,1	62	42,5	100	47,6			243	43,2
Master and PhD	29	14,0	17	11,6	27	12,9			73	13,0

Source: Own elaboration, based on 563 respondents. *p-value <0.01; ** *p*-value <0.001

The geographical origin of the sample showed a predominance of European tourists (57.5%), which increased to 68.6% in the summer of 2020. This variable is of great importance in this analysis, since tourists have different levels of thermal comfort depending on their geographical origin, an analysis that is already well documented in the literature (e.g., Lindner-Cendrowska & Błażejczyk 2018; Lopes et al. 2021b).

## Results

### Microclimatic measurements

In this study, we conducted microclimatic measurements and surveys during the summer of 2019, the winter of 2019-2020, and the summer of 2020. We present the results of these measurements in Appendix 2, highlighting the main meteorological parameters.

It is observed that the mean air temperature (Mean Air Temperature – AT) varied significantly between the study periods, reaching 18.4°C in winter, 27.8°C in summer 2019, and 25.3°C in summer 2020. Calibrated wind measurements (Wχ) showed predominantly light breezes, with an average of 1.0 m·s^-1^. However, we highlight an exception on December 28, 2019, during the winter, when wind intensity reached 6.0 m·s^-1^. Relative humidity (RH - %) values ranged from 33.6% to 84.2% throughout the study periods. These data help to understand the meteorological conditions during the administration of surveys to tourists.

### Influence of clothing and physical activity on tourists’ Outdoor Thermal Comfort (OTC)

The choice of clothing during travel varies significantly among different types of tourists. In this study, AT and Icl of tourists were assessed using 1.0°C intervals to understand these variations (Fig. 2). During the summers of 2019 and 2020, the observed range of Icl values was between 0.43 and 1.49. It's noteworthy that no linear regression relationship between AT and Icl was identified in either season, with r^2^ ≤ 0.1. Additionally, the critical point marking the inflection in tourists' Icl occurs at 21.7°C.

**Fig. 3:**
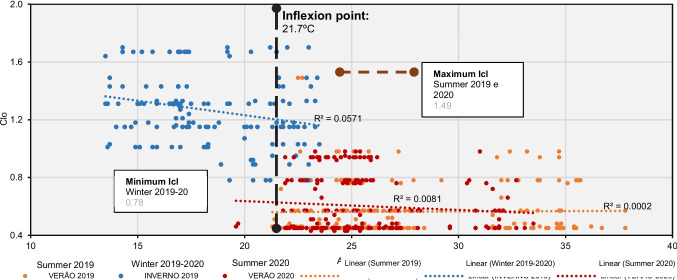
Relationship between AT (ºC) and clothing insulation (Icl) in summer 2019, winter 2019-2020 and summer 2020 Source: Own elaboration, based on 563 questionnaire surveys of tourists and microclimate measurements

Figure 3 illustrates the inflection point in the relationship between the average Icl values used by tourists (mIcl) and AT at 1.0°C intervals. The connection between these variables is evident through the regression line. Although most respondents were surveyed under AT conditions ranging from 22.0°C to 27.0°C, there is a clear correlation between the increase in AT and the reduction in clothing layers, as expressed by mIcl (r^2^ = 0.846; *p*-value < 0.05).

**Fig. 4:**
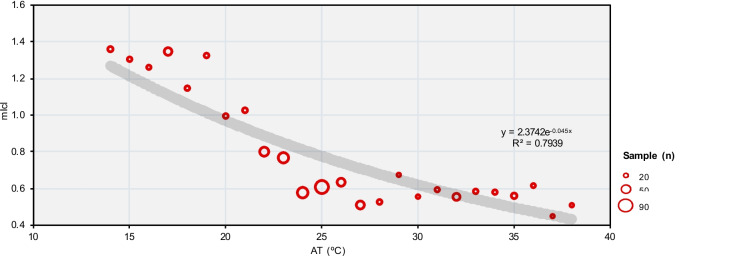
Relationship between AT (ºC) and average clothing insulation (mIcl). Source: Own elaboration, based on 563 questionnaire surveys of tourists and microclimate measurements

Contrary to expectations that national and international tourists would exhibit significantly different mIcl values, it was observed that both groups showed a similar relationship between Icl and AT. International tourists generally perceive Portugal's climate as mild, leading them to wear slightly less clothing than their national counterparts. Additionally, when AT exceeded 24.0°C, both national and international tourists opted for lower Icl (Fig. 4).

The primary reason behind this trend is that as AT increases, tourists tend to choose lighter clothing sets to maintain appropriate levels of OTC during their travels in the tourist destination.

As advocated by Xi et al. (2020), tourists tend to make more deliberate choices regarding the number of clothing layers they wear during their visits to destinations compared to residents. This suggests that tourists' clothing decisions are more calculated and influenced by factors specific to their travel experience. Nonetheless, it is imperative to consider cultural differences in Icl. In this case, we must not forget that most of the visitors are from Western countries (European continent = 75.3%, see Table 3), which does not allow us to assess with great accuracy the cultural differences related to the clothing inherent in certain cultures, although it can be noted that significant differences were found between European and non-European groups (*p*-value < 0.05).

**Fig. 5:**
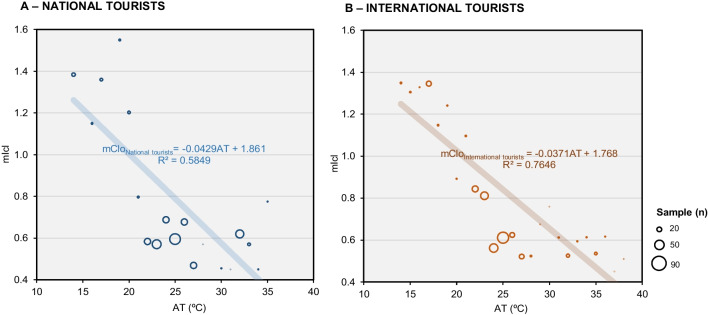
Relationship between AT (ºC) and average clothing insulation (mIcl) for domestic and international tourists (**A**) Domestic tourists; (**B**) International tourists. Source: Own elaboration, based on 563 questionnaire surveys of tourists and microclimate measurements

Examining the relationship between Icl and the gender of respondents, as depicted in Fig. 5, reveals that both male and female tourists exhibit similar behavior in the relationship between Icl and AT (r^2^ = 0.740 and r^2^ = 0.733, respectively, with a *p*-value < 0.05). This implies that gender does not significantly alter the pattern of clothing choices concerning OTC in tourists.

**Fig. 6:**
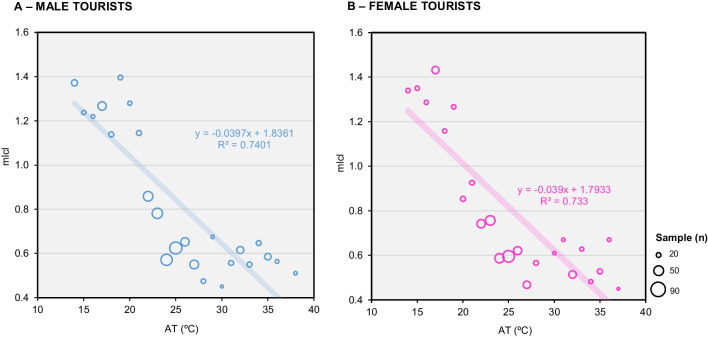
Relationship between air temperature (ºC) and average clothing insulation (mIcl) for tourists according to gender. **A** Male tourists; (**B)** Female tourists. Own elaboration, based on 563 questionnaire surveys of tourists and microclimate measurements

The thermal insulation of the preferred clothing was correlated with the weather conditions of the 2019 and 2020 summer and 2019-2020 winter seasons, and with the gender variable (Fig. 6).

The thermal insulation of clothing ranged from 0.4 Icl, which was the minimum value, to 1.75 Icl, which was the maximum value. It should be noted that among the group of tourists, especially in summer, female individuals wore fewer layers of clothing (Icl < 0.5). In general, individuals wore clothing between 0.4 and 1.25 Icl in summer, as opposed to winter, where the minimum insulation was 0.76 Icl.

The functional group of the elderly also showed, on average, higher Icl (mIcl = 0.86). This trend was observed in all seasons but was more pronounced in the winter of 2019-2020 (mIcl = 1.33). Icl was also calculated for tourists' mean thermal sensation values (mTSV). The relationship between Icl and thermal sensation generally showed an inverse relationship between thermal sensation and number of layers in summer (> mTSV ↔ < mIcl) and a relationship between the two variables in winter (< mTSV ↔ > mIcl). In summer, respondents chose a thermal sensation between warm and very warm (85.0%) and mostly wore clothing with a Icl of less than 1.00 (81.6%). On the other hand, clothing with a mIcl greater than 1.25 was mostly worn in winter and for a TSV < -1 (72.9%).

**Fig. 7:**
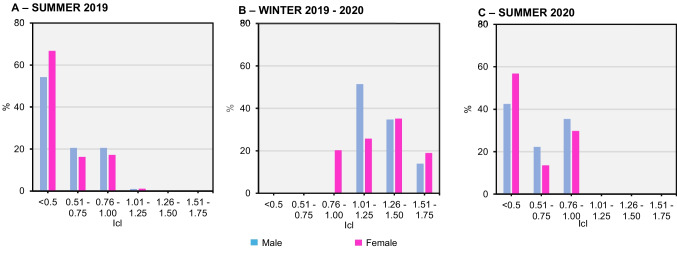
Thermal insulation of clothing (Icl) worn by tourists in relation to season and gender (**A**) Summer 2019; (**B**) Winter 2019-2020; (**C**) Summer 2020. Source: Own elaboration, based on 563 questionnaire surveys of tourists and microclimate measurements

Figure 7 shows the average activity level of tourists in 60-minute intervals between 10:00 AM GMT+00 and 5:00 PM GMT+00 during the summer of 2019, the winter of 2019–2020, and summer of 2020. The following questions were used: *B.1. How would you rate your current thermal sensation based on your current cloth?* and *B.7. Please identify the clothing you are currently wearing.*

**Fig. 8:**
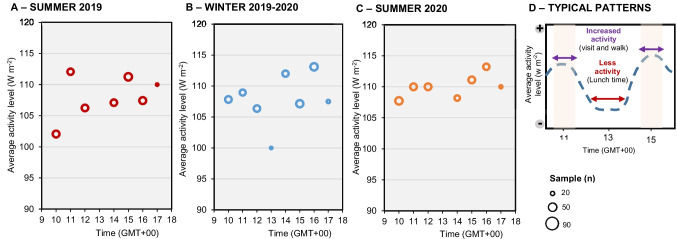
Relationship between the average level of activity (W/m^2^) and the time of day when tourists were surveyed (**A**) Summer 2019; (**B**) Winter 2019-2020; (**C**) Summer 2020; (**D**) Typical time patterns. Source: Own elaboration, based on 563 questionnaire surveys of tourists and microclimate measurements

There is no clear relationship between the time and the average activity level. Nevertheless, it seems that the activity level was higher during the time intervals from 10:30 GMT+00 to 11:30 GMT+00 and between 14:30 GMT+00 to 16:00 GMT+00 (Fig. 8). This suggests that tourists plan their visit times well during the day, and the lunchtime period corresponds to the time with the lowest tourist activity. It is noteworthy that, generally, the afternoon hours slightly increase the average activity level.

The average activity level of tourists corresponding to AT ranges of 1.0°C was calculated. Only during the winter season of 2019–2020 was there a relationship between AT and the average activity level, indicating that tourists engaged in more intense activities when AT was lower (Fig. 8). This relationship can be expressed by the linear regression line through Equation 1:

1 $$\text{AAL}=-\text{0,6385AT}+\text{120,53}\left({\text{r}}^{2}=\text{0,9943}\right)$$

During the summer, a weak correlation was observed between AT and the average activity level of tourists, suggesting that intraurban movement and activity levels were rarely influenced by the thermal environment. Furthermore, the average activity level of surveyed tourists in the summer of 2019 (107.8 W.m^-2^) was lower than in the winter of 2019–2020 (109.2 W.m^-2^). Nevertheless, these values were lower in both periods compared to the average activity level in the summer of 2020 (110.0 W.m^-2^). These results highlight that: (i) tourists prefer engaging in more physically demanding activities during winter to keep warm in response to lower AT conditions; (ii) the effects of COVID-19 during the summer of 2020 increased movement between locations, possibly due to perceived risks by tourists who felt uncomfortable or unsafe in the same place for longer periods.

## Discussion and conclusions

The evaluation of Icl and activity level as one of the six physiological factors that determine human OTC is a relevant reference of this paper, especially in the context of Porto (Portugal). The results of this research indicate that the clothing worn by tourists in different periods and seasons is significantly related to climatic and meteorological conditions. During the summer, tourists tend to wear lighter clothing, with a significant reduction in the number of layers of clothing as the temperature increases (*r*^2^ = 0.846; *p*-value < 0.05). This behavior is consistent for both national and international tourists, suggesting a similar adaptation to the mild climate of Portugal and Porto in particular.

The analysis also showed that, on average, women wore fewer layers of clothing than men during the summer (Icl < 0.5), indicating possible differences in OTC perception between the gender. This is consistent with a generally greater ability to sweat in men of the same age and physical condition as women, which would allow for better thermoregulation in hot weather (Hazelhurst & Claassen 2006). Recent studies found that females tend to exhibit higher thermal acceptability and are more accepting of thermal conditions compared to males (Huang et al. 2019; Lam et al. 2018). Zafarmandi et al. (2024) found gender differences in the study they applied to Theran (Iran), but it is worth noting that Bröde and colleagues' research in southern Brazil found no significant effect of gender on thermal perception (Bröde et al. 2012), although they did observe that men tended to wear more clothing in warm conditions than women, similar to our study (*r*^2^ = 0.740 and *r*^2^ = 0.733, respectively, with a *p*-value < 0.05).

In addition, the elderly tended to wear more layers of clothing, especially in winter. In the winter of 2019-2020, tourists aged 65 and older had an mIcl = 1.33, while adults had an mIcl = 1.23 during the same period. The relationship between thermal insulation of clothing and thermal sensation suggests that in summer, tourists adjust their clothing to ensure OTC, while in winter, the relationship is more complex and may depend on additional factors. Analyzing the study by Xi et al. (2020), where the activity level of tourists was higher in winter, in our case study, the average activity level of the surveyed tourists in summer 2019 (107.8 W.m^-2^) was lower than in winter 2019-2020 (109.2 W.m^-2^). However, the average activity level was higher in summer 2020 (110.0 W.m^-2^).

In terms of physical activity, the results show a seasonal variation, with higher physical activity in summer. This can be attributed to the milder and more pleasant weather, which encourages tourists to engage in more outdoor activities.

Compared to other studies, our results are consistent with previous studies showing the impact of Icl on OTC (Tian et al. 2022; Xi et al. 2020; Zafarmandi et al. 2024). However, this effectiveness varies across different locations, populations, and cultures. This underscores the importance of considering regional variations in clothing habits and cultural influences. For instance, residents of Phoenix may not face social restrictions on clothing choices, while those in Marrakech adhere to cultural traditions dictating more covered attire, even in warm weather. Indeed, cultural factors play a critical role in clothing choices, as demonstrated by studies showing differences in clothing preferences and thermal tolerance between genders and across cultural contexts (Zafarmandi et al. 2024), such as among Chinese female visitors and Italian outdoor space users (Tung et al. 2014; Salata et al. 2018). The study by Zafarmandi et al. (2024) has the peculiarity of being influenced by hijab restrictions, which affect thermal comfort levels where tourists may not want to wear so much clothing. The same is not true in a case like that of Portugal, which differs in the conditions presented in Tehran (Iran). These findings highlight the need for nuanced considerations of cultural influences and gender differences in understanding and predicting OTC perceptions.

The research highlights the importance of tourists' adaptation of clothing and physical activity to meteorological conditions, which directly affects OTC during the tourism activities. The findings presented in this study have significant implications for the design of tourist spaces, suggesting the need to consider not only local weather conditions, but also the adaptive behavior of tourists in terms of clothing and physical activity. In fact, from the analysis carried out, it appears that tourists adjust the clothes they wear throughout the day and vary their activities to mitigate the effects of higher temperature variations.

## Limitations

During the conduct of this study on OTC conditions in urban contexts, particularly in the tourism sector, several limitations were identified. Thus, it is essential to acknowledge these limitations to contextualize the results and provide a transparent view for future studies. Among the main limitations, the following are outlined:

- Some literature review on OTC and clothing characteristics, but the importance of considering cultural differences in clothing use, acclimatization, and level of physical activity – despite the relevance of OTC and the influence of clothing on the tourist experience, there is a gap in the literature regarding studies that thoroughly address these aspects in specific tourist contexts. The impact of this relationship on tourist satisfaction is lacking in in-depth and targeted studies, as tourists are often exposed to unknown and sometimes adverse outdoor thermal conditions during relatively short periods of stay.
- Impact of body movement – the influence of body movement on modifying air velocity and thermal insulation is not comprehensively addressed in any of the cited studies evaluating the relationship between physical activity and the insulation of clothing. The absence of specific data on how body movement can affect ventilation and insulation effectiveness is a gap that deserves additional attention.
- Lack of assessment of clothing worn in extreme heat conditions in tourism – Our study acknowledges the omission of specific assessments conducted under extreme heat conditions, despite recognizing the importance of such investigations. The absence of data in this scenario limits the generalizability of results to situations of intense heat. Typically, temperatures of 37°C (considered extreme conditions) were reported, and ISO standards suggest appropriate clothing for extreme conditions and work periods based on activity levels. There exists literature on various types of clothing utilizing both passive and active methods to mitigate extreme conditions. However, our study underscores the unique characteristics of tourist activities, which may render individuals less sensitive to extreme heat. Additionally, cultural considerations and the use of clothing with distinct properties must not be overlooked.
- Absence of parameters for face masks – the lack of specific thermal insulation parameters for face masks is recognized. Despite their use by some individuals, especially during the COVID-19 period but also in areas more susceptible to air pollution, the lack of data on the thermal properties of these accessories limits the accuracy of OTC assessments.

## Future research

These limitations highlight specific areas that deserve attention in future research. Given the rapidly evolving circumstances, it is crucial to adapt research methods to address these limitations and understand OTC conditions in tourism areas, particularly those related to leisure practices in urban outdoor environments. In future studies, the team is interested in evaluating a comparison with the same methodology in other territorial areas, but above all in conducting an analysis based on the following factors:

- To evaluate how different levels of physical activity and body movements affect ventilation and the insulation provided by clothing. This research could include controlled experiments that measure changes in air velocity and thermal insulation capacity due to movement, thus filling the existing gap on the relationship between physical activity and clothing insulation that we were unable to accurately explore in this study.
- To analyze the variations caused by cultural variables (e.g., the use of the hijab) or the use of face masks (in destinations with high air pollution) on the thermal sensation in different climatic contexts. This study could include data collection in specific regions, in Portugal and in other different territorial contexts, also using thermal mannequins to measure the impact of cultural clothing and face masks on the sensation of thermal comfort.

## Appendix 2

Table 6

**Table:** Average weather conditions during the questionnaire surveys of tourists

Study period		_AT_ (°C)	Wχ (m.s^-1^)	RH (%)	G_RAD_ (W.m^-2^)	T_MRT_ (°C)
Summer 2019	Average	27.8	0.90	56.1	598.8	49.8
	Median	26.2	0.80	54.3	601.4	50.5
	Minimum	21.3	0.00	34.5	31.3	34.0
	Maximum	37.8	3.85	81.3	896.5	64.7
Winter 2019–2020	Average	18.4	0.88	46.2	249.5	28.1
	Median	17.8	0.65	42.9	243.6	27.0
	Minimum	13.5	0.00	34.3	21.2	8.1
	Maximum	23.5	6.04	69.3	556.8	48.4
Summer 2020	Average	25.3	1.07	62.8	594.1	48.5
	Median	24.8	0.83	64.8	598.3	49.9
	Minimum	19.6	0.00	33.6	31.9	34.9
	Maximum	33.5	4.10	84.2	845.4	60.4
Total	Average	24.4	0.96	56.0	482.3	43.7
	Median	24.3	0.78	56.1	483.5	48.2
	Minimum	13.5	0.00	33.6	28.3	8.1
	Maximum	37.8	6.04	84.2	765.4	64.7

Source: Own elaboration, based on 563 questionnaire surveys of tourists and microclimate measurements

## Abbreviations

AMP: Área Metropolitana do Porto
AT: Air Temperature
CFD: Computational Fluid Dynamics
EU: European Union
G_RAD_: Global Radiation
Icl: Clothing Insulation Value
ISO: International Organization for Standardization
mTSV: Mean Thermal Sensation Values
NbS: Nature-Based Solutions
OTC: Outdoor Thermal Comfort
RH: Relative Humidity
SBS: Shadow Behavior Simulations
SD: Standard Deviation
SPSS: Statistical Package for the Social Sciences
SVF: Sky View Factor
T_MRT_: Total Mean Radiant Temperature
TSV: Thermal Sensation Vote
Wx: Wind Speed
